# The sexual and reproductive health needs, experiences and challenges faced by women in Saudi Arabia from stakeholders’ perspectives

**DOI:** 10.3389/fgwh.2025.1491689

**Published:** 2025-09-17

**Authors:** Noura Alomair, Samah Alageel, Nathan Davies, Julia V. Bailey

**Affiliations:** 1 Community Health Sciences Department, College of Applied Medical Sciences, King Saud University, Riyadh, Saudi Arabia; 2 Centre for Psychiatry and Mental Health, Institute of Population Health, Queen Mary University of London, London, United Kingdom; 3 Research Department of Primary Care and Population Health, University College London, London, United Kingdom

**Keywords:** muslim women, women's health, cultural norms, Saudi arabia, sexual and reproductive health, qualitative research

## Abstract

The perceptions, beliefs, and experiences of Muslim women regarding sexual and reproductive health are influenced by numerous factors. This study explores the sexual and reproductive health needs and challenges faced by women in Saudi Arabia, drawing on insights from key professional stakeholders. It examines the challenges faced by Saudi women and the obstacles they encounter in accessing healthcare services from the perspectives of stakeholders.

**Methods:**

The study employed a qualitative design using semi-structured one-on-one interviews with healthcare professionals, policymakers, and religious scholars in Riyadh, Saudi Arabia. The data were analysed using reflexive thematic analysis. Participants were recruited using purposive sampling.

**Results:**

The professional stakeholder sample included 16 participants: seven healthcare providers, six policymakers, and three religious scholars. The data revealed widespread gaps in women's sexual and reproductive health knowledge, including misconceptions about menstruation, reproduction, contraception, and sexual function. Participants described how cultural taboos and restrictive social norms foster negative attitudes toward sex and sexuality, which contribute to misinformation, fear, and sexual dysfunction. Women's ability to seek information or express sexual needs was often limited by expectations of modesty, with sexual awareness perceived as inappropriate or shameful. Stakeholders highlighted childhood sexual abuse as a prevalent yet silenced issue, with lasting effects on sexual health and well-being. Stakeholders emphasised the importance of improving sexual health education, promoting public awareness, and encouraging open, culturally sensitive dialogue to support sexual and reproductive health.

**Conclusion:**

This study highlights the impact of societal taboos and cultural beliefs on women's sexual and reproductive experiences. The study emphasises the need for improved education, greater public awareness, and open communication to address misconceptions and support women's well-being. Promoting women's sexual well-being includes creating a more informed, inclusive, and supportive environment for women's sexual and reproductive health and advocating for policies that protect and promote their sexual and reproductive health.

## 1 Introduction

Sexual and reproductive health is a crucial aspect of public health and has a significant impact on individuals' well-being (1). It encompasses the right to access reproductive health services, access to comprehensive information and services that support optimal sexual health. This includes the prevention and treatment of sexually transmitted infections (STIs), promoting safe and consensual sexual relationships, and enabling individuals to make informed decisions regarding their reproductive choices (1, 2). In discussions about reproductive health, there is often a significant focus on women's reproductive health. This emphasis is because women and girls have specific health requirements that result from certain conditions that only women go through, which might have some negative health implications for them. These conditions, including pregnancy and childbirth, are not in themselves diseases but normal physiological and social processes that most women experience that can carry health risks and require medical care (3).

In the past decade, there have been significant improvements in the sexual and reproductive health and rights of women worldwide, including lower maternal mortality rates and better access to contraception and HIV treatment (4, 5). However, there are still significant gaps and inequalities in sexual and reproductive health worldwide (4, 6). For example, over 200 million women lack access to modern contraception, especially in marginalised communities (7). Maternal mortality is still the second leading cause of death among girls aged 15-19, and one in three women experiences physical or sexual violence in their lifetime (5, 7). These disparities are particularly concerning for Muslim women, who often face additional cultural, religious, and socio-economic barriers that restrict their access to essential sexual and reproductive health services (8). This exacerbates the existing inequalities and underscores the urgent need for targeted interventions (8).

Religious and cultural factors play a significant role in shaping people's sexual health beliefs and practices (8, 9). In Muslim communities, women's ignorance of sexual and reproductive health topics is viewed positively as a symbol of modesty and purity (8, 9). In the absence of comprehensive sex education, this can lead to poor health practices, limited knowledge, misinformation, and myths surrounding sexual health (8). Lack of sexual health knowledge can have negative consequences on health, such as unintended pregnancy, sexually transmitted infections, unsafe abortions, and psychological and emotional distress (9, 10). Lack of understanding about sexuality may be linked with feelings of shame, guilt, anxiety, and sexual dysfunction, all of which can impact physical and mental well-being (11).

The Kingdom of Saudi Arabia is deeply influenced by conservative Islamic traditions and social norms (12), which have a significant impact on women's sexual and reproductive health (8). The cultural context, religious beliefs, and legal frameworks create a complex environment that can affect women's sexual and reproductive health (8). This can impact access to information and healthcare services, as well as their ability to make independent decisions regarding their bodies and sexual lives. Since 2016, Saudi Arabia has been experiencing societal changes and extensive reforms. These changes have significantly impacted women's lives, including improved access to sexual and reproductive health information and services, leading to substantial improvements in their quality of life (13, 14). However, multifaceted factors shape Saudi women's sexual and reproductive health beyond existing laws and regulations. Understanding the complex factors that influence Saudi women's sexual and reproductive health is essential for policymakers, healthcare providers and public health advocates. Research is needed to shed light on the factors that shape Saudi women's experiences and explore the implications for their overall sexual well-being. This study aimed to examine Saudi women's sexual health experiences, needs, and the challenges generated from the perspectives of policymakers, healthcare providers, and religious scholars in Saudi Arabia.

## 2 Methods

Ethical approval was obtained from the UCL ethics committee with Reference no. 10157/001 and King Fahad Medical City in Riyadh with Reference no. FWA00018774.

### 2.1 Study design

The study is a qualitative design using semi-structured one-on-one interviews. Qualitative research was chosen because it provides valuable and unique understandings that quantitative methods cannot address. Most research done on sexual health topics in Saudi Arabia is in the quantitative field (15–17), which can be insufficient when exploring a sensitive and complex subject such as sexual and reproductive health.

### 2.2 Sampling and recruitment

Purposive sampling was used to identify professional stakeholders. We chose stakeholders based on their professional involvement in activities related to women's health, health education, sexual and reproductive health, and religious studies. A stakeholder is defined as an individual or group with a substantive interest in an issue, including those with some role in policy-making positions (18).

We recruited healthcare professionals, including health educators, obstetricians/gynaecologists, and psychologists. We contacted policy-making agencies and health sector officials directly via telephone or email invitations to recruit stakeholders. We also recruited respected and influential religious leaders and scholars from various universities in Riyadh, Saudi Arabia.

Riyadh was chosen as the study site because of its crucial role in shaping national healthcare policy, service provision, and public discourse in Saudi Arabia. As the capital city, Riyadh is home to major government institutions, academic centres, and the largest public and specialist hospitals in the country. These institutions serve patients from across the Kingdom, making Riyadh a hub for diverse healthcare experiences and perspectives. Additionally, the city hosts key stakeholders involved in the health, education, and policy sectors, providing strategic access to participants who have direct influence on the development and implementation of sexual health-related programs.

### 2.3 Data collection

The participants were given the choice of face-to-face interviews at a location of their choice or telephone interviews. All face-to-face interviews took place in a private room to ensure confidentiality. Fourteen stakeholder interviews were conducted face-to-face, and interviews with two religious scholars were conducted via telephone due to logistical challenges.

The lead author (NA), a female public health researcher from Saudi Arabia with experience in qualitative research, conducted the interviews. Before the interview began, stakeholders signed a consent form and completed a brief demographic questionnaire to provide descriptive data. The questionnaire included questions about their job description, expertise, and affiliated institutions (Table 1).

**Table 1:** Stakeholders’ detailed sample characteristics.

Professional role	Speciality/field
Programme director of public health department	General practitioner
Programme director of public health department	Family medicine consultant
Head of governmental hospital	OBGYN consultant
Head of public health department	Medical sciences—Health education
Head of clinical education unit	Medical sciences—Health education
Healthcare General Directorate	Medical sciences—Health education
OBGYN consultant	Specialises in infertility
OBGYN consultant	Specialises in infertility
OBGYN consultant	Specialises in high-risk pregnancies
OBGYN consultant	Specialises Urogynaecology
Psychologist	Relationship and marriage counsellor
Health educator	Specialises in women's health
Health educator	Specialises in antenatal care
Academic scholar—Islamic studies	Teaching and researching religious views on family planning and contraception
Academic scholar—Islamic studies	Teaching and researching “Jurisprudence of Family”
Academic scholar—Islamic studies	Teaching and researching marriage from a religious perspective

Participant recruitment continued until data saturation was achieved (19), at which point no new themes, concepts, or insights emerged from the interviews. Saturation was assessed iteratively during data collection through regular review of transcripts and coding. By the 14th interview, the research team observed that the data had become repetitive and no novel codes were being generated. Two additional interviews were conducted to confirm saturation, after which recruitment was concluded at a total of 16 participants.

Most of the interviews were in Arabic, and one was in English. All interviews were audio-recorded with the participant's consent, each lasting between 30 and 90 min.

### 2.4 Topic guide

The research team created separate topic guides for different stakeholder groups, which were informed by a systematic review of the literature on the factors influencing Muslim women's sexual and reproductive health (8). The ecological model of health behaviour guided the development of the interview questions (20). The model suggests that health is influenced by personal, social, cultural, economic and political factors (20).

For healthcare providers, questions explored the types of sexual and reproductive health issues commonly encountered in clinical settings, patient attitudes toward sexual and reproductive health, and providers' comfort and responsibilities in discussing sexual and reproductive health topics. Example questions included: “What types of SRH cases do you encounter?” and “How do you feel about discussing sexual health with patients?”

For policymakers, the guide focused on current challenges in addressing women's sexual and reproductive health, existing programs, and the feasibility of implementing sexual and reproductive health education in public health strategies. Sample questions included: “Do you know of any programs that focus on sexual and reproductive health for women?” and “What are the challenges to introducing formal sexual and reproductive health education in Saudi Arabia?”

For religious scholars, questions examined religious perspectives on sexual and reproductive health education, societal views on discussing sexual health, and the potential role of religious leaders in shaping community attitudes toward these issues. Example questions included: “What are Islamic views on teaching sexual education?” and “Should sexual and reproductive health education be addressed solely from a religious viewpoint or include scientific content as well?”

Across all guides, participants were also asked about attitudes toward including sexual health education programs, barriers to sexual health communication, and suggestions for improving public awareness and education.

### 2.5 Data analysis

The data were analysed using a reflexive thematic analysis (21). All interviews were audio recorded and professionally transcribed verbatim, and the lead author (NA) reviewed them alongside the recordings to ensure accuracy. NA coded all interviews using ATLAS.ti, and SA independently coded a sample. The codes were then discussed and compared, and differing opinions were resolved through discussions.

The interviews were conducted and transcribed in Arabic. Data analysis was conducted in the original Arabic to preserve the authenticity and cultural nuances of the participants' narratives. Selected quotes were translated into English after the analysis for reporting purposes, along with additional quotes not included in the manuscript, to allow non-Arabic-speaking team members to engage with a broader portion of the data.

Two bilingual authors (NA and SA) independently translated the quotes and reached consensus on the final versions. To further ensure translation accuracy, a back-translation process was employed for selected quotes. Additionally, several full transcripts were translated into English to facilitate theme validation and interpretation by non-Arabic-speaking members of the research team.

We used an inductive approach to develop codes and themes, consistent with Braun and Clarke's reflexive thematic analysis (21). Coding was data-driven, and categories were iteratively grouped to construct an analytical framework and identify preliminary themes. The research team continuously reviewed and refined the themes to ensure they accurately reflected the coded data and the dataset as a whole. The quotes presented in the findings were independently translated from Arabic to English by two authors (NA and SA), who then jointly agreed on the final translation.

We attempted to enhance rigour by offering evidence from the data to support all interpretations. We examined outlier cases and contradictory data, compared data within and between cases in the dataset, and compared the findings with those of other studies (22).

## 3 Results

The stakeholder sample consisted of 16 participants, 12 females and four males. We interviewed seven healthcare providers: five obstetricians/gynaecologists (OBGYN) or urogynaecologists of both genders and two female health educators. Additionally, six policymakers from various health organisations, including both male and female representatives, and one female and two male religious scholars, were interviewed. Table 1 provides an overview of stakeholders' characteristics. Three main themes emerged from the data, including sexual and reproductive knowledge and misconceptions, sexual and reproductive health views and experiences, and societal expectations of women's sexual and reproductive health.

### 3.1 Sexual and reproductive knowledge and misconceptions

Stakeholders explained that Saudi women lack sexual and reproductive health knowledge in areas including puberty, menstruation, basic human anatomy, reproduction, contraception, pregnancy, childbirth, and sexually transmitted infections.

“They don't know their cycle, what’s going on with their body, what’s the processes and stages from puberty to menstruation to reproduction. These topics are like a grey area or no-no area.” **(S12, Policymaker)**

This lack of awareness may result in a lack of understanding of what sexual intercourse entails, unfamiliarity with the reproductive organs responsible for sperm production or misinformation, such as the belief that male infertility is impossible.

“We have a significant lack of understanding of what constitutes sexual intercourse and what is considered common knowledge in other parts of the world. We often depend on knowledge passed down from our mothers and grandmothers. What I see most in my field of work is a lack of basic understanding of sexual intercourse. For example, some women do not know what an orgasm is or do not understand what sexual dysfunction is.” **(S11, Urogynaecologist)**

The presence of blood after the first sexual intercourse was still considered proof of virginity for many people. Misconceptions about what is expected from women after their first sexual experience, such as spotting, put women at risk of severe consequences, including physical and emotional harm.

“We need to educate people about the first night [first sexual intercourse]. For example, I once saw a friend who was newly married. He called me. He was very distraught and told me that the first time, he didn’t see any spotting or blood. So, he immediately thought that it was something serious [his wife is not a virgin]. So, I told him I need to see you; I need to talk to you face-to-face. This is very serious. He said he read this information on a blog, that if your wife doesn’t have any spot of blood, it means she is not a virgin. And he was a very educated man…” **(S8, OBGYN)**

### 3.2 Sexual and reproductive health views and experiences

#### 3.2.1 Negative views toward sex and sexuality

Women in Saudi Arabia are typically brought up in a culture where sex and sexuality are seen as shameful, immoral, and even illegal outside of marriage. For some, the idea of sex, even within marriage, is difficult to accept, leading to significant physical and psychological challenges.

“This topic [sex] for young girls, even in their own home, is a no-no. It’s a no-go area. You shouldn’t talk about it or discuss it with anyone until she is married, at this time her mother might or might not talk to her about this topic just for her sake because she is getting married… but when you grow up with this being no no and Eib [shameful] and Haram [forbidden], how can you expect her at 25 years old for example to suddenly change her entire belief system and accept the idea of having sex with her husband. How can this suddenly be okay when I grew up with it being wrong and forbidden, and taboo? Things cannot change overnight.” **(S12, Policymaker)**

Healthcare providers explained that many women in Saudi Arabia are experiencing sexual health issues that are directly influenced by their conflicting attitudes toward sex. Conditions such as vaginismus are a major sexual health concern for women in Saudi Arabia. Healthcare providers mentioned that many women suffer in pain and guilt for years without seeking help, and many are unaware of their health conditions and whether they are treatable.

“Many women are expected to accept having sex in the first night of marriage, even if she didn’t know her husband well. Sadly, women are usually timid, and some will require psychological help to overcome difficulties in having sexual intercourse. It would be best if you worked with her… because vaginismus does not require surgical intervention. It’s all about helping women accept their sexual needs and be comfortable with their husbands because there is something deep-rooted in them that makes having sex unacceptable.” **(S8, Female, OBGYN)**

Healthcare providers said that women may lack sexual pleasure and fulfilment in their marital relationships. For many women, sexual satisfaction is not a priority in marriage, as the primary purpose of sex is often seen as procreation. Any sexual activity beyond procreation is typically solely for the fulfilment of the husband's sexual needs. Additionally, women lack knowledge about their bodies and are unaware that sex can be pleasurable for them.

“I have seen many patients come to me after 10 or 20 years of marriage. And sex is a source of stress and fear for them. She never enjoys sex, and she isn’t even aware that sex can be pleasurable. Sexual relationships are just for the husband, for having children or for what is expected of her by society. She never enjoys a sexual relationship with her husband; she is miserable, and this has a huge impact on her mental health. Our society places no significance on sexual pleasure for women; it is not a priority in our society.” **(S13, Female, Psychologist)**

Healthcare providers attributed some of the sexual health challenges experienced by women to the social consequences of extramarital sex, which can be severe for women. Participants mentioned that women might be at risk of physical harm or even death. When extramarital relations among men were discussed, no consequences other than STIs were mentioned.

“If an unmarried girl gets pregnant, she will be labelled as bad for the rest of her life, and it is a very depressing situation. If an illegal pregnancy happens, the outcome for women is very severe. They might commit suicide, run away from home if their family finds out, be subjected to violence, or even killed due to the conservative nature of society.” **(S7, Male, Policymaker** **+** **OBGYN)**

#### 3.2.2 Sexual abuse in childhood

Stakeholders emphasised that childhood sexual abuse is a prevalent and severe issue that affects both genders. Sexual abuse is a complex topic to discuss, and most victims keep their abuse to themselves. Child molestation is often associated with feelings of great shame, guilt, and confusion. Victims tend to blame themselves for being sexually abused and are generally afraid that they will be punished and stigmatised for the rest of their lives. Stakeholders pointed out that most cases involve being sexually abused by someone they know, such as a family member, relative, or close family friend. This makes speaking up about the abuse even more difficult, as they fear negative consequences for themselves and their abuser.

“It [sexual abuse] could happen from another student, a boy who took advantage of a young girl, a neighbour, I mean, sadly, it could come from a brother, a cousin, someone in the family. It might leave them unable to have a healthy sexual relationship, and their marital relationship would be deeply affected. The trauma that they went through needs to be dealt with professionally, but sadly, that never happens. They need someone to help them through this trauma, but they will never speak up.” **(S3, Female, OBGYN)**

Sexual abuse can have a significant impact on a victim's understanding of healthy sexual relationships and may lead to sexual dysfunction in adulthood. Physicians often do not uncover instances of childhood sexual abuse in their patients until it has significantly affected their mental health, relationships with their partners, and even their relationships with their children. Many victims are unaware of the connection between their sexual problems in adulthood and their experiences of sexual abuse in childhood.

“It’s [sexual abuse] a big issue, especially in schools, and maybe I hear a lot about it because of my work. Some people can overcome its effects. They would bury it deep inside; it would be like something locked up inside of them, and they would never talk about it. But for some people, it affects them. It has an impact on their life. They will not be able to live a normal life ever again.” **(S3, Female, OBGYN)**

### 3.3 Societal expectations of women's sexual and reproductive health

#### 3.3.1 Modest women are shy and uninformed

Religion, traditional gender roles, and social expectations dictate that women should be unaware and uninterested in their sexual health. This expected ignorance was specific to women. Women's understanding of sexual health and sexuality was considered inappropriate, and being aware was associated with being promiscuous. At times, women were thought to pretend to be ignorant to conform to societal norms of purity and modesty.

“**S2:** it is difficult for a woman to ask about these things. A lot of people believe it is Eib even to discuss this in front of women, even if they are adults over 18 years of age. Commonly, they won’t talk to her about it or teach her about these topics until she is married with kids. After that, they would discuss things with her openly, but before that, it was considered Eib and not up for discussion.

**NA:** Considered Eib by whom?

**S2:** It's the society's views, not a religious one. It is associated with society; religion accepts discussing these topics.” **(S2, Female, Health educator)**

A physician specialising in infertility and IVF treatments saw a female patient who had been struggling with fertility issues for many years and had been seen by several doctors over the years. After many consultations, it was discovered that she and her husband had never had complete sexual intercourse. The physician revealed that this issue is prevalent, and she frequently encountered patients with similar problems.

“I once had a patient not too long ago; she had been married for five years. She came to us as an infertility case, and we asked her about her sexual life. She said everything was good and there were no problems there. And then we discovered they had never had sex in the five years of their marriage. And she went through many fertility doctors until she came to us.”. **(S3, Female, OBGYN)**

A policymaker mentioned an educational program designed to increase sexual health awareness among youth in schools. This program was only offered to male students. When asked why females were excluded from the program, she explained that it is more accessible and acceptable for males to receive sex education.

“**NA:** Why did you choose to target boys, not girls?

**S9:** To be honest, the choice wasn't made by me when I attended the meeting, and [colleague name] said that we would start with boys’ schools, then we might consider it for the girls' schools.

**NA:** Was there any rationale for this choice?

**S9:** No, I don't know, maybe because it is easier to offer the programme to boys.” **(S9, Female, Policymaker)**

#### 3.3.2 Communications and discussions about sexual health

The societal norms and taboos surrounding discussions of sexual matters make it challenging for women to express their sexual desires or openly communicate with their husbands about sexual issues. Women fear being judged as impure or as having had previous sexual experiences if they express any level of awareness or have any sexual expectations. Their shyness and inability to communicate their sexual needs to their spouses also impede their sexual fulfilment and pleasure.

“Women might be afraid that men would think she wasn't raised properly if she talked about her sexual knowledge. She fears her husband will ask her how she knows these things. The man expects her to be shy and unaware. ‘I don't know, that’s just how we [society] are.”. **(S13, Female, Psychologist)**

The lack of communication between partners can have a significant impact on women's sexual and reproductive health. For instance, a patient undergoing chemotherapy experienced a lot of pain and severe health consequences because she was unable to communicate with her husband that she was unable to engage in sexual intercourse.

“I had a patient who had cancer and was on chemotherapy. The chemotherapy was affecting her body and her health and made her very ill. It affected sexual intercourse between her and her husband. Her husband did not understand her situation; he wanted his needs and must get them. And this was damaging her health physically. But when did she decide to talk? When she got so ill that she had to be hospitalised.” **(S5, Female, Health educator)**

Many participants voiced frustration over the societal taboos surrounding sexual and reproductive health topics, particularly in the context of communication between partners. Experts in psychology and women's health suggested that many issues could be resolved through open communication between partners. Stakeholders emphasised the importance of addressing taboos related to sexual and reproductive topics to promote open communication among women.

“It is important for women to realise that expressing their desires and needs is okay, just like everything in life. Talking about your needs with your husband is not shameful or forbidden! It is not shameful!” **(S13, Female, Psychologist)**

#### 3.3.3 Gender inequality and traditional gender roles

There were conflicting opinions and views on how women should behave before and within marriage, as well as what is expected of them in a marital relationship. Some participants showed judgment toward women who are shy and have trouble accepting the idea of sex within marriage. It was also observed that women are expected to please their husbands, regardless of their personal problems.

“Most of the time, the husband is waiting for the wife to initiate; it is because the man is too polite and out of respect for his wife, he waits for the wife to show interest and show him that she wants him. This puts him in a very uncomfortable situation, making him feel rejected and unwanted. Why would you [the woman] make him feel this way?!” **(S13, Female, Psychologist)**

In recent years, there has been a shift in traditional gender roles in Saudi society, with women becoming more independent and empowered. Traditionally, women were expected to bear the sole responsibility of preserving the marriage and protecting it from divorce. The empowerment of women was cited as one reason for the increase in divorce rates, as women have become “impatient” and “unwilling to compromise.”

“We need to educate women on how to build a home for their family and what the wife’s responsibilities are. Sadly, this came naturally in our mothers and grandmothers’ generations; they didn’t need anyone to teach them how to become responsible. They [new generation women] fail to understand life’s priorities and responsibilities. And that’s why divorce rates are high now. Nowadays, they would sacrifice their marriage so easily and end their marriage over something so silly… she [women] needs someone to educate her on what is expected from her in her home, with her husband, how to maintain the stability of their life and the importance of marriage.” **(S3, Female, OBGYN)**

Religious scholars believe that a lack of communication between partners, excessive openness, and having work colleagues of the opposite gender are contributing factors to the recent rise in divorce. Religious scholars also stated that women being “unreasonably” dissatisfied with marriage and younger generation women developing “strong and stubborn personalities” are among the main reasons for the rise in divorce rates.

“Sadly. Now, with the recent increase in openness, what has happened is that strong personalities have developed in both genders, and this affects the relationships between them. They [women] become stubborn and do not compromise… Also, with social media like Snapchat, which I think is the number one cause of divorce, girls tend to compare their relationships with what they see on social media and make them have unrealistic expectations, which can ruin marriages.” **(Religious scholar 2, Male)**

## 4 Discussion

According to our research, professional stakeholders observed that Saudi women often hold deep-rooted negative views toward sex and encounter challenges in changing these perceptions within the context of marriage. This contributes to the challenges women face in expressing their needs and discussing sexual matters with their partners. Societal expectations of modesty and lack of sexual education also prevent women from seeking healthcare services for sexual health issues. There are conflicting opinions on women's behaviours before and within marriage, often leading to judgmental views toward sexual knowledge among women.

The sexual health of Muslim women is deeply influenced by a complex interplay of values, traditions, and experiences that shape their perceptions towards sexual and reproductive health (8, 9, 23). Family, culture, religion, and upbringing all play significant roles in this regard (9, 23). Cultural and traditional values instilled from childhood can profoundly impact women's perceptions of their sexuality, sexual needs, and attitudes towards sex (9, 23). In many Muslim cultures, sex outside of marriage is considered immoral and even illegal in some countries. Previous research underscores the challenges faced by Muslim women in feeling unprepared for their first sexual experiences and encountering difficulties in accepting the concept of sex, even within marriage (24). This can lead to various physical and psychological problems, such as emotional distress, anxiety, and sexual dysfunction (25–27). Therefore, it is crucial to provide comprehensive sex education that includes discussions on consent, sexual pleasure, contraception, reproductive health, and healthy relationships. Integrating sexual health education into premarital counselling, school curricula, and community outreach programs can create sustainable channels for delivering accurate information. Equipping women with this knowledge encourages critical thinking and can empower them to make informed decisions about their sexual health (28).

Religious and cultural values often dictate societal norms, expectations, and rules regarding women's sexuality. Cultural beliefs can foster harmful gender stereotypes, such as the notion of women's virginity before marriage (29–31). While virginity is highly valued in Islam, particularly in the context of premarital chastity, the social perception of sex, especially women's sexuality, can often be negative and associated with impurity in some communities (30, 32). In line with our findings, many communities perceive the presence of blood as proof of a woman's virginity (33). This misconception can be very harmful for women, as the social consequences of extramarital sex are much more significant and dangerous for them.

Premarital sex, specifically, losing virginity or unintentional pregnancy, is one of the leading causes of suicide and death for young Muslim women worldwide (34). Many of these deaths are concealed honour killings, and many of these murders go unpunished (35). Honour killings continue to occur globally, with an estimated 5,000 women and girls being killed every year in the name of honour by their family members (36, 37). These killings are often carried out due to perceived offences related to female sexuality, such as engaging in extra-marital or premarital relationships (35). While the term “honour killing” is widely used in global discourse, it has been critiqued for its Orientalist and essentialist undertones, which risk framing gender-based violence as a uniquely “cultural” problem of specific regions (38, 39). It is therefore important to understand these acts within broader, transnational structures of patriarchy and gender-based violence that exist across societies. While this research focused on women's health, men's knowledge and behaviour significantly impact women's sexual and reproductive health, particularly regarding decision making, sexual relations and reproduction. Future research should therefore incorporate the perspectives of men to provide a more comprehensive understanding of these dynamics.

Addressing gender inequality requires challenging deeply rooted cultural beliefs, promoting gender equality, and fostering open sexual health dialogue in the home, school, and healthcare settings. Collaboration with religious leaders is crucial, as faith-aligned messaging can improve acceptability and counter misconceptions. We recommend engaging religious leaders in discussions on gender equality, as this is vital for promoting positive change within Muslim cultures. Emphasising the Islamic principles of justice, compassion, and respect for women's rights has the potential to facilitate a more inclusive and equitable understanding of gender and sexuality.

According to our research, cultural taboos present a significant obstacle to open sexual health communication within Muslim communities. It is widely believed that discussing sexual health outside of marriage is prohibited by Islamic teachings (40). This belief can create a barrier to accessing sexual healthcare services and can prevent couples from seeking the help they need (8). Our study participants, healthcare providers in particular, underscored the crucial role of open communication in cultivating marital satisfaction, allowing partners to express their sexual needs, preferences, and concerns openly. Research shows that insufficient knowledge about human anatomy and sexual pleasure can contribute to sexual dysfunction and dissatisfaction (41–43). A study focusing on premarital education for Muslim couples revealed that participants emphasised the need for education on sexual pleasure and relationships, mainly because these topics are often not formally taught or openly discussed among couples (44, 45). To ensure cultural relevance and feasibility, we recommend integrating sexual health education into existing premarital counselling frameworks already mandated in Saudi Arabia, with training for healthcare providers to address sexual health topics sensitively and effectively. Additionally, partnerships with religious scholars can support public messaging that aligns with Islamic principles while promoting open dialogue and informed decision-making. These approaches may help bridge the gap between cultural expectations and the evolving needs of Saudi women.

### 4.1 Strengths and limitations

A significant strength of this study is the diversity of professional stakeholders in our sample. It allowed us to gather a wide range of expertise and perspectives on the sexual and reproductive health of Saudi women. By interviewing various stakeholder groups, we were able to explore the views of individuals who are not typically represented in the literature on Saudi women's sexual and reproductive health, such as religious leaders, who wield significant influence over public opinion. It allowed us to capture the perspectives of those responsible for implementing and shaping policies and services, including policymakers and healthcare providers. However, it's important to note that the views expressed by the stakeholders in this study are representative of those who are enthusiastic about improving women's sexual and reproductive health in Saudi Arabia and endorse the importance of providing sexual and reproductive health services in the country.

This study was conducted in 2019, before several major legal reforms affecting women's rights in Saudi Arabia. As such, the findings reflect the sociocultural and legal context of that time. While participants primarily discussed cultural and religious influences on sexual and reproductive health, we recognise that legal and policy frameworks also play a critical role. These dimensions were not a central focus of this study and warrant further exploration in future research.

Although this study focuses on Muslim women in Saudi Arabia, we acknowledge that this term encompasses a wide range of experiences, shaped by differences in social class, education, region, and personal beliefs. The terminology used reflects participants' own framing and the cultural-religious context of the study, but is not intended to suggest a homogeneous group. Reporting findings from a specific group can unintentionally imply that certain experiences are universally shared, when in reality, considerable variation exists both within and across cultures and religions (46). As Arousell and Carlbom argue, future research should move beyond generalised portrayals of Muslims as a single group and recognise religious heterogeneity and individuals’ capacity to interpret and negotiate religious teachings (47).

Conducting interviews in Arabic enabled the participants to communicate confidently and use local idioms, euphemisms, and natural expressions. However, some cultural references may not have been easily translated, resulting in a potential loss of meaning. To mitigate this, the translation of selected quotes was conducted independently by two bilingual researchers, followed by a consensus discussion to ensure semantic accuracy. Nevertheless, the translation process can be analytically valuable and a critical challenge that can aid interpretations (48, 49).

In conducting a reflexive thematic analysis, we acknowledge that data interpretation is inherently shaped by the researchers' perspectives, backgrounds, and positionalities. The research team included bilingual Saudi researchers with academic and professional experience in sexual and reproductive health, which helped in contextualising the data within the local sociocultural framework. To manage potential biases, we engaged in ongoing, reflexive discussions throughout the analysis process to critically examine how our assumptions, values, and positionalities might influence the development of themes. Regular debriefings were held to challenge interpretations and ensure that the coding process remained grounded in participants' narratives. This collaborative and reflective approach helped enhance analytical rigour and trustworthiness of the findings.

## 5 Conclusion

Our research highlights significant gaps in sexual and reproductive health knowledge among Saudi women. Societal taboos and cultural beliefs have a considerable impact on women's sexual and reproductive experiences. The study emphasises the importance of awareness, support, and open dialogue in addressing these issues and working toward a more inclusive and understanding society. Promoting women's sexual well-being includes ensuring their access to quality healthcare services and advocating for policies that protect and promote their sexual and reproductive health.

## Data Availability

The datasets presented in this article are not publicly available due to concerns about participant confidentiality. Requests for access may be directed to the UCL Research Ethics Committee at ethics@ucl.ac.uk.
